# A model-based approach to generating annotated pressure support waveforms

**DOI:** 10.1007/s10877-022-00822-4

**Published:** 2022-02-10

**Authors:** A. van Diepen, T. H. G. F. Bakkes, A. J. R. De Bie, S. Turco, R. A. Bouwman, P. H. Woerlee, M. Mischi

**Affiliations:** 1 Department of Electrical Engineering, Eindhoven University of Technology, Eindhoven, 5612 AZ The Netherlands; 2 Catharina Hospital, Eindhoven, The Netherlands

**Keywords:** Patient-ventilator interactions, Asynchronies, mechanical ventilation, Model based methods, Machine learning

## Abstract

Large numbers of asynchronies during pressure support ventilation cause discomfort and higher work of breathing in the patient, and are associated with an increased mortality. There is a need for real-time decision support to detect asynchronies and assist the clinician towards lung-protective ventilation. Machine learning techniques have been proposed to detect asynchronies, but they require large datasets with sufficient data diversity, sample size, and quality for training purposes. In this work, we propose a method for generating a large, realistic and labeled, synthetic dataset for training and validating machine learning algorithms to detect a wide variety of asynchrony types. We take a model-based approach in which we adapt a non-linear lung-airway model for use in a diverse patient group and add a first-order ventilator model to generate labeled pressure, flow, and volume waveforms of pressure support ventilation. The model was able to reproduce basic measured lung mechanics parameters. Experienced clinicians were not able to differentiate between the simulated waveforms and clinical data (P = 0.44 by Fisher’s exact test). The detection performance of the machine learning trained on clinical data gave an overall comparable true positive rate on clinical data and on simulated data (an overall true positive rate of 94.3% and positive predictive value of 93.5% on simulated data and a true positive rate of 98% and positive predictive value of 98% on clinical data). Our findings demonstrate that it is possible to generate labeled pressure and flow waveforms with different types of asynchronies.

## Introduction

Mandatory positive pressure mechanical ventilation is a form of life support. It is difficult to optimize the ventilator settings for a patient and ventilator-induced lung injury remains a major concern. When there is a spontaneous breathing effort, support modes may be used whereby the patient can control tidal volumes. During the pressure support ventilation (PSV), the patient triggers each breath and the ventilator supports this effort by a positive pressure during the inspiration phase. Mismatches between the patient’s effort and the mechanical ventilator support are called asynchronies. A high rate of asynchronies is associated with adverse outcomes such as discomfort, higher work of breathing, and an increased mortality rate [8, 19]. Asynchronies are underdiagnosed because the detection, classification, and resolvement of these asynchronies are challenging for bedside intensive care unit (ICU) clinicians, even for the more experienced ones. Besides, continuous monitoring of mechanical ventilation is not feasible in clinical practice.

There is a clinical need for real-time decision support and optimization of PSV. Reliable automatic asynchrony detection using machine learning techniques could be a first step towards this goal. Studies have already been conducted on the automated detection of asynchronies [19]. Especially neural networks provide an interesting opportunity to develop algorithms for real-time detection of a wide variety of asynchronies [5, 39], however, there are also studies that take a model-based approach [12]. A bottleneck for studies is the amount of available labeled clinical data to improve the training, testing, and comparing these machine learning algorithms. Although labeled datasets exist [34], they are often not publicly available, recorded in a single-center or only specific asynchronies are labeled. It is therefore difficult to compare different machine learning techniques. Moreover, manual labeling and advanced monitoring, such as esophageal pressure or neural activity monitoring, are required in obtaining such a dataset, which is time-consuming, operator dependent, and prone to errors. Some types of asynchronies are only scarcely available in clinical datasets, and cannot be easily recreated due to the higher risks it might impose to the patient.

Our goal was to generate a synthetic, automatically labeled dataset containing pressure, flow, and tidal volume curves of a diverse ICU population during PSV to augment incomplete or a small clinical dataset. Augmenting a dataset with synthetic data to help training and testing of machine learning algorithms is well known in other fields [1]. We made use of well-known, validated lumped element models of the lung, and extended these with a simple ventilator model to model breaths during PSV. To check whether this approach is successful, we perform several tests on the synthetic data, including a test with experienced clinicians and state-of-the-art machine learning.

## Material and methods

We take the following approach in this paper:

- We implement a non-linear lung-airway model of the lung. We optimize this model for various pulmonary conditions, such as obesity, acute respiratory distress syndrome (ARDS), chronic obstructive pulmonary disease (COPD) and idiopathic fibrosis (fibrosis). We hypothesize that the heterogeneity of these lung pathologies can be incorporated in this model of low complexity to be able to simulate mechanical ventilation waveforms in sufficient detail. We combine this lung-airway model with a new simplified ventilator model.
- The complete model is implemented in a circuit simulator, LT Spice [26], which is a commonly used open-source circuit simulator. The simulator is able to handle the non-linear components in the model. The model communicates with a MATLAB-script that is able to generate the muscle input wave and to post-process and store the simulated data.

In order to check whether this approach is successful, we perform several tests on the synthetic data: (1) We check whether the lung model is able to replicate the measured lung mechanics parameters of the different lung conditions; (2) whether the waveforms correspond to clinical data; (3) whether experienced clinicians are able to distinguish the simulations and clinical data; (4) we perform several tests with a machine learning algorithm that had a high performance on clinical data [5].

### Patient model

For the patient model, we select the validated model of Athanasiades [3]. This model is similar to the model of Bates [6], but it includes turbulence, nonlinear relationships, airway collapse, and visco-elastic properties.

**Fig. 1:**
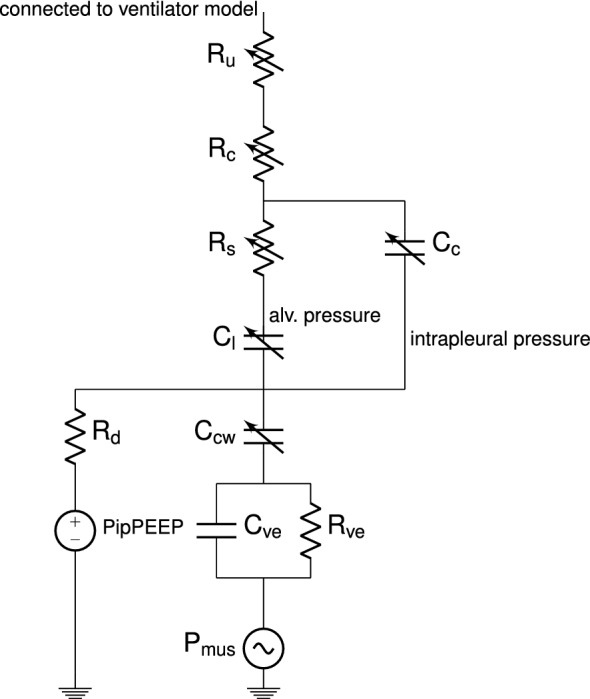
The lung model which is an adapted version of [3]. Note that the components are nonlinear and are coupled with each other. Compared to the model of Athanasiades [3], the Kelvin body and chest wall have been switched and an extra resistor $R_d$ and a voltage source $\mathrm {PipPEEP}$ are added to ensure correct initial conditions for the intrapleural space pressure.

The model of Athanasiades was chosen because it is a validated model for maximumum effort tests with parameters that can be partly re-used. It can model expiratory flow limitation using a collapsible resistance and compliance, which is an important feature in COPD patients and patients suffering from obesity. Moreover, since non-linear equations are used to account for the lung and chest wall compliance, the effect of applying PEEP and inspiratory pressure is modeled more realistically, it is also required to model fibrosis, ARDS, COPD and obese patients properly. Viscous damping is an important feature in pressure waves, and therefore needs to be included in the model. The model includes turbulence in the upper airways which is especially an important feature for obese patients.

More complex models are available that also include these effects. However, clinical parameters are often estimated using one-lung models, and it is therefore convenient to use a one-lung model, such that these parameters can be re-used.

Given all the above requirements, the model complexity and number of parameters therefore seems reasonable.

The patient model (Fig. 1) consists of three variable resistances modeling the upper airways ($R_u$), collapsible airways ($R_c$), and small airways ($R_s$). The resistance of the upper airway $R_u$ is given by a nonlinear flow-dependent Rohrer resistor, which accounts for turbulence:

1 $$\begin{aligned} R_u = A_u+K_u \mid \dot{V}_{cw}\mid ,\ \end{aligned}$$

where $A_u$ is the linear resistance of the upper airways, $K_u$ is a constant, and $\dot{V}_{cw}$ the airflow rate.

The resistance of the collapsible airway $R_c$ varies with the volume of the collapsible airway segment $V_c$, and is given by:

2 $$\begin{aligned} R_c=K_c(V_{cmax}/V_c)^{2} ,\ \end{aligned}$$

where $K_c$ is a constant and $V_{cmax}$ is the maximum volume of the collapsible airways.

The resistance of the small airways $R_s$ captures the dependency of the small airway resistance on the alveolar volume $V_A$ [3]:

3 $$\begin{aligned} R_s = A_s e^{K_s(V_A - RV)/(V^*-RV)}+B_s ,\ \end{aligned}$$

where $A_s$, $K_s$, $B_s$, and $V^*$ are constants and *RV* is the residual volume of the lung.

The variable capacitor $C_c$ models the compliance of the collapsible airway segment. Athanasiades et al. [3] modeled this by a piece-wise continuous function. To make the implementation in a circuit simulator possible, we replace this function by a twice differentiable function, which has the same (sigmoidal) shape in the physiological region:

4 $$\begin{aligned} V_c = \frac{V_{cmax}}{(1+e^{-A_c(P_c-B_c)})^{D_c}} \ , \end{aligned}$$

where $V_{cmax}$ is the maximum volume of the collapsible airways, $A_c$, $B_c$, and $D_c$ are patient dependent constants, and $P_c$ is the pressure over the capacitor $C_c$.

$C_{cw}$ is the compliance of the chest wall. The volume of the chestwall $V_{cw}$ is modeled by a sigmoid function [3]:

5 $$\begin{aligned} V_{cw} = \frac{TLC-RV}{0.99+\exp {\frac{-(P_{cw}-A_{cw})}{B_{cw}}}}+RV \ , \end{aligned}$$

where *TLC* is the total lung capacity (the maximum value of the sigmoid function), *RV* the residual volume (the minimum value of the sigmoid function), $P_{cw}$ the pressure in the chest wall, and $A_{cw}$ (the shift of the curve) and $B_{cw}$ (related to the slope of the curve) are patient dependent constants.

For the alveolar volume $V_A$, we replace the original curve with the exponential curve determined empirically by Venegas et al. [36], which describes the normal lung and different disease archetypes:

6 $$\begin{aligned} V_A = \frac{A_l}{(1+e^{-B_l(P_t-D_l)})} \ , \end{aligned}$$

where $A_l$, $B_l$ and $D_l$ are patient dependent constants and $P_t$ is the transmural pressure. Together with $C_l$, the linear $C_{ve}$ and $R_{ve}$ form a nonlinear Kelvin body that mimics the visco-elastic properties of the lung and chest wall. $C_{ve}$ and $R_{ve}$ are constants and are chosen such to mimic available literature. Note that the order of the Kelvin body and chest wall compliance is switched as compared to Athanasiades et al. [3]. This is done to ensure that lung and chest wall volumes are equal during simulation.

**Fig. 2:**
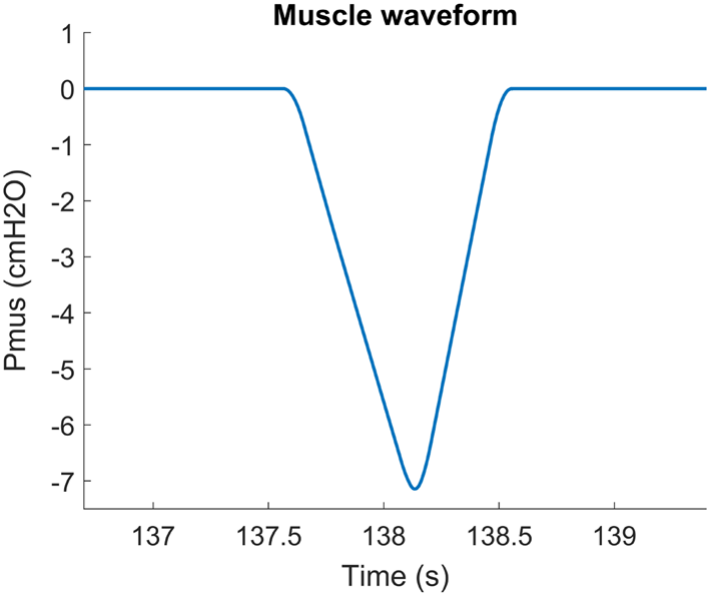
An example of a typical muscle waveform for one breath. We use a rounded trapezoid with a different slope for the rising edge and falling edge to model the breath

Finally, $\mathrm {P_{mus}}$ models the effect of the respiratory muscle activity. We use a rounded trapezoid with a different slope for the rising edge and falling edge (Fig. 2), which is similar to measured patient muscle effort. We also added a sine wave to this signal, with an amplitude between 0.25 and 1 $\hbox {cmH}_2$O and a frequency between 1 and 2 Hz, to model the cardiac oscillations often observed in ventilator waveforms.

We have added a voltage source $\mathrm {PipPEEP}$ and a very high resistance $R_d$ to ensure correct initial conditions of the equal lung and chest wall volume and to avoid floating nodes.

### Tuning of the lung model parameters

Athanasiades et al. [3] use measurements of four different healthy test subjects to fit four parameter sets. We take this set of parameters as our baseline. To create a diverse dataset with different types of critically ill patients, we have selected the following types of patients: Obese patients, patients suffering from ARDS, COPD patients, and patients with idiopathic fibrosis. By using available clinical data in the literature, we propose the following changes in the lung mechanics as compared to the “healthy” archetype:

- In obesity, due to closure of the peripheral airways and due to the diaphragm being pressed towards the head in supine position [28], the TLC and functional residual capacity (FRC) are lower than in healthy subjects. Since obese subjects generally breathe in a lower lung volume range than their healthy counterparts, the total compliance at FRC is reduced. We use the relationship found in Pelosi et al. [30] based on the body mass index (BMI) to calculate a value for the total compliance ($C_{tot}$). The upper airway resistance is strongly increased mostly due to increased turbulence [10, 28, 29, 38].
- ARDS patients have a loss of oxygenated lung tissue caused by inflammation of the lungs, fluid accumulation in the lungs, and a partial collapse of the lung (atelectasis). A smaller part of the lung has normal mechanical properties (the baby lung). Taking everything into account, the lungs in ARDS patient are more heterogenous with a low compliance compared to the healthy lungs [20, 31]. This results in a smaller residual volume, functional residual capacity, and total lung capacity. The volume in the lung is generally decreased compared to healthy. Resistances of the airways are moderately increased compared to a healthy individual [14]. We use measured values for the resistance and compliance of ARDS patients to create the new parameter sets.
- The term COPD is used for all patients with non-reversible obstructive airflow. However, there exists great variation between the different phenotypes of COPD [24]. For this work, we have made a selection of common changes that are found in COPD patients as compared to the healthy archetype. COPD is characterized by high airway resistance and low lung elastic recoil [15, 18]. This results in high compliance of the lung tissue, which in turn results in higher lung volumes. For this reason, the total lung capacity (TLC), residual volume (RV), and functional residual capacity (FRC) are all higher than in a healthy person [27]. The severity of COPD is indicated by the ratio RV/RV normal and TLC/RV. The RV is found by multiplying the predicted residual volume of a healthy person based on height and age found in [35] by 1.7. The inspiratory capacity (IC) is lower as compared to a healthy person. The expiratory resistance is much higher in patients with COPD due to excessive central airway collapse especially during expiration [25]. The small airway resistance is also higher than the healthy patient archetype, caused by the loss of elastic recoil, that normally opens the small airways.
- In idiopathic fibrosis, the lung tissue is very stiff and has low compliance. The residual volume, functional residual capacity, and total lung capacity are all lower compared to healthy individuals [22, 32]. The airway resistance is slightly lower than in healthy individuals.

Based on these descriptions, we compose a set of requirements for every patient archetype (Table 1). We hand-tune the parameters of the model to meet these requirements, and represent the different disease conditions, creating a more diverse set of parameters. The parameter sets are available in the supplementary information.

**Table 1:** Requirements for volume, resistances, and compliances per patient archetype as compared to healthy

	Healthy	Obese	ARDS	COPD	Idiopathic fibrosis
RV	–	Smaller	Much smaller	1.7*Predicted RV healthy	Smaller than healthy
FRC (ZEEP)	–	0.33*FRC healthy	0.4*Healthy	Larger than healthy	Smaller than healthy
TLC	–	RV + IC	RV + IC	Two times RV	Smaller than healthy
$R_{insp}$	–	Larger than healthy	Larger than healthy	7.5	The same as healthy
$R_{exp}$	–	Larger than healthy	Larger than healthy	15	The same as healthy
$C_{tot}$ (at FRC)	–	233.3*exp[– 0.086*BMI] + 40 [30]	Smaller than healthy	0.075	Smaller than healthy

**Fig. 3:**
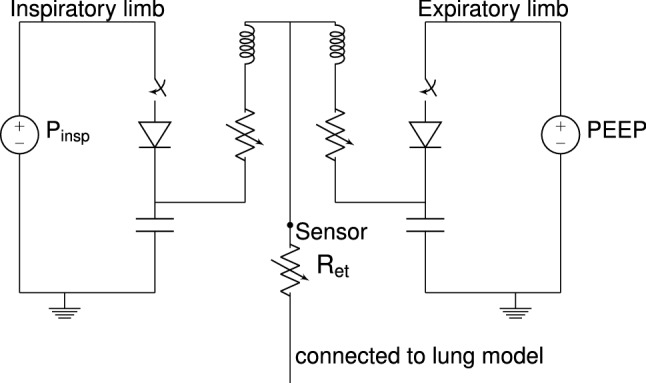
Equivalent circuit of the ventilator model. Note that the endotracheal tube and the resistances modeling the tubing system are not simple resistances, but are modeled by Rohrer’s equation and depend on the flow through the elements

### Ventilator model

The ventilator model is shown in Fig. 3. The node below $R_{et}$ is connected to the wire above $R_{u}$ in Fig. 1.

The ventilator model consists of the following parts:

- The endotracheal tube $R_{et}$ (the tube which is inserted in the trachea through the mouth during invasive ventilation), is modeled as a variable resistance to include turbulent airflow. The endotracheal tube adds an extra resistance $R_{et}$ in series with the airway resistances from the patient. We choose the same approach as Flevari et al. [16], where the resistance of the endotracheal tube is modeled by Rohrer’s equation similar to the upper airway resistance. Flevari et al. measure the parameters for different types of endotracheal tubes, we use the data for the 9 mm endotracheal tube.
- The tubing system of the breathing set connects the endotracheal tube and the ventilator. This system is split into an inspiratory circuit and an expiratory circuit. Both are modeled by an inductor (inertance), capacitor (compliance) and resistor, Fig. 3. Turbulent flow is important for typical flow rates and leads to a large flow rate dependent resistant that is modeled using Rohrer’s equation. Wenzel et al. [37] measure the resistance for different brands and types of tubes. We take the same approach and use their measured values. The inertance and compliance of these breathing tubes are also measured by Wenzel et al. [37] and are also included in the model. The parameters of the tubing are available in the supplementary information.
- The ventilator itself consists of two pressure sources and different unidirectional valves. The ventilator pressurization is modelled by two voltage sources that represent the pressure sources in the ventilator. They operate at the expiratory pressure (PEEP) and the inspiratory pressure $P_{insp}$. The valves are modeled by switches and by unidirectional diode-like elements, which block inspiration from the contaminated expiratory connection. Valves are opened and closed when the ventilator is triggered and when it cycles. This results in a block wave or a trapezoidal wave for the ventilator. Tables with parameters of the ventilator are available in the supplementary information.

The pressure and the flow in ventilated patients are usually measured at the proximal location after the Y-piece but before the endotracheal tube, this is also where we measure the pressure at the airway opening (labeled ’sensor’ in Fig. 3). The effect of the parasitics of the tubing is very important for modeling the specific waveform characteristics measured at the sensor.

### Asynchronies

During PSV, the ventilator triggering (delivery of $P_{insp}$) and cycling (switching from $P_{insp}$ to PEEP) are initiated by the patient. If the patient starts inspiration, air starts flowing into the lung and the pressure at the sensor becomes lower than PEEP. Triggering can therefore be achieved by putting a threshold on the flow or the pressure. Cycling is usually done when the flow reaches a percentage of the peak flow (usually 10-80 percent). Ideally, the ventilator would trigger and cycle exactly at the same time as the patient’s start and end of inspiration effort. If the trigger and cycle timings differ too much from the inspiration time of the patient, we are observing an asynchrony.

To classify whether a breath is an asynchrony or a regular breath, the time between the start of patient inspiration and ventilator triggering (start-inspiration delay) and the end of patient inspiration and ventilator cycling (end-inspiration delay) need to fall into certain margins. We employ the same margins as Bakkes et al. [5]:

- A normal breath has an end-inspiration delay larger than – 100 ms and smaller than 300 ms. The start-inspiration delay must be lower than 250 ms. All other breaths are asynchronies.
- Early cycling: the ventilator cycles too soon. More specifically, we define that the end-inspiration delay must be shorter than – 100 ms (the ventilator cycles more than 100 ms before the end of patient inspiration).
- Late cycling: the ventilator cycles after the end of patient effort. More specifically, we say that the end-inspiration delay must be longer than 300 ms for a breath to be late cycling.
- Delayed inspiration: There is a significant trigger delay between the patient inspiration and the ventilator inspiration; the ventilator inspiration triggers late compared to the patient inspiration. The start-inspiration delay is longer than 250 ms.
- Ineffective effort: patient effort is not followed by a ventilator pressurization. In other words, there is a patient effort but the ventilator is not triggered, the start-inspiration delay and end-inspiration delay are therefore not defined.

Since delayed inspiration occurs during triggering, and late cycling and early cycling occur during cycling, also combinations of delayed inspiration and early cycling, and delayed inspiration and late cycling can be present during one breath.

In clinical data, the start and end of patient inspiration are usually difficult to determine precisely without an esophageal balloon manometry. In the simulation, the patient and ventilator are fully controlled. We know exactly when patient inspiration started and ended and when the ventilator triggered and cycled, which creates sets of completely annotated waveforms.

### Validation

Before generating the waveforms, we check whether the model parameters are tuned correctly according to Table 1. We compare $R_{insp}$, $R_{exp}$, compliances, and lung volumes in the model to the values reported in the literature. RV and TLC can directly be observed from the model parameters. FRC can be calculated by calculating the volume at $P_t+P_c= -P_{cw}$. We define $R_{exp}$ and $R_{insp}$ as the sum of $R_u$, $R_c$, and $R_s$ during expiration and inspiration at low flow. $C_{tot}$ is calculated by taking the slope of the combined lung-chest wall pressure–volume curve of the model at FRC. We report the values using zero end-expiratory pressure (ZEEP), except for COPD where we report the values using PEEP and $P_{insp}$.

After this initial verification, a synthetic dataset with more than 60.000 breaths was created, using the implementation of the model in LT Spice XVII [26]. The disease type of the patient was randomly selected. The input muscle waveform $\mathrm {P_{mus}}$ is generated by MATLAB R2019b [23] using the parameters in Table 2.

**Table 2:** Parameters for the muscle effort ($\mathrm {P_{mus}}$) used to create asynchronies

	Amplitude (cmH2O)	Fall time (s)	Rise time (s)
Normal breath	5–10	0.25–0.35	0.5–0.7
Early cycling	5–10	0.25–0.35	0.7–0.9
Late cycling	5–10	0.25–0.35	0.5–0.7
Delayed inspiration	3.5–4.5	0.25–0.35	0.7–0.9
Ineffective effort	1.7-2.2	0.4–0.6	0.4–0.6

Typical values for the breathing rates, and trigger and cycling thresholds are reported in Table 3. PEEP and $P_{insp}$ are chosen in such a way that the tidal volume is 0.4-0.6 L during a normal breath.

**Table 3:** Breathing rate, cycling threshold, trigger threshold during the simulations

Breathing rate	12–18 Breaths (min)
Cycling thresholds	10–80 percent of peak flow
Trigger threshold	1–2 cmH2O below PEEP

To ensure balanced classes and enough training examples for the machine learning algorithm, each class of asynchronies has the same number of breaths. The asynchrony type of each breath is easily retrieved since the timing of the patient and ventilator are saved. Low-pass filtered white noise (bandwith 15 Hz) with a signal-to-noise ratio (SNR) between 15 and 16 was added to the pressure and an SNR between 30 and 31 to the flow.

Although we started with 60.000 breaths, the method can quickly generate more breaths. On a single core of an Intel Core i7 7th Gen, the program runs 4.7 times real-time (including the initialization of the muscle waveform in MATLAB). In other words, for 10 s of simulation, 2.1 s of execution time is required.

Validation of the obtained dataset is not a trivial task. Therefore we include a variety of tests from airway and lung parameters, evaluation of the waveforms by experienced clinicians and using an existing validated neural network model.

The authors visually compared the synthetic waveforms to clinical data that were obtained from patients after cardiac surgery [13]. The local Institutional Review Board (R16.054) associated with Catharina Hospital Eindhoven approved the use of patient-related waveforms for medical research. The clinical data contained all asynchronies, which were also visually compared to the asynchronies in the synthetic data.

A survey was sent to 16 experienced clinicians from two different instituations to check whether they were able to differentiate the simulated waveforms and the previously mentioned clinical waveforms. The clinicians assessed 32 samples, which were 16 clinical and 16 synthetic waveforms. Both 16 clinical waveforms and 16 synthetic waveforms were randomly selected from both datasets. The first 16 samples were the same for every clinician. The last 16 samples were unique for every clinician. The clinicians determined whether the provided waveforms were a simulated waveform or a clinical waveform. To statistically test whether they were able to differentiate the two, Fisher’s exact test was used, for which a $p < 0.05$ was considered to be significant. Fisher’s exact test tests whether the difference between two proportions in a 2 $\times$ 2 contingency table is significant.

As a last test, we apply the neural network trained on clinical data proposed in Bakkes et al. [5] to the synthetic dataset. In the orignal paper [5], the machine learning algorithm was trained and tested on the same (small) labeled clinical dataset. It is therefore not known how well this algorithm generalizes when applied to a different dataset. The algorithm is a one-dimensional version of the U-net architecture and detects the start and end of patient inspiration in unlabeled pressure, flow, and volume waveforms when esophageal pressure is not available. The network consists of four contracting and expanding layers and is trained using the adam optimizer in conjuction with categorical cross-entropy as a loss function [5]. 4275 labeled clinical breaths were used to train the network using 300 epochs. The 60.000 synthetic waveforms were only used for validation.

Since we know the true timing of the synthetic dataset, we report the true positive rate (TPR), positive predictive value (PPV), and root-mean-squared error (RMSE) of the algorithm, which are defined in the following way:

7 $$\begin{aligned} \text {TPR}= & {} \frac{\text {{ Patient efforts correctly identified}}}{\text {All patient efforts present in data}} \,, \end{aligned}$$

8 $$\begin{aligned} \text {PPV}= & {} \frac{\text {{ Patient efforts correctly identified}}}{\text {All identified patient efforts}} \,, \end{aligned}$$

9 $$\begin{aligned} \text {RMSE}_{\text {start}}= & {} \sqrt{\frac{1}{n}\sum _{i=1}^{n}\left( S_i-\hat{S}_{i} \right) ^2} \,, \end{aligned}$$

where $S_{i}$ is the true start time of patient inspiration and $\hat{S}_{i}$ is the estimated start time by the machine learning algorithm.

10 $$\begin{aligned} \text {RMSE}_{\text {end}} = \sqrt{\frac{1}{n}\sum _{i=1}^{n}\left( E_i-\hat{E}_i \right) ^2} \,, \end{aligned}$$

where $E_{i}$ is the true end of patient inspiration and $\hat{E}_{i}$ is the estimated end time by the machine learning algorithm.

This test, including all its presented evaluation metrics (Eq. 7–10) gives us information both about the ability of the algorithm to generalize and about the quality and abnormalities of our synthetic dataset.

## Results

### Basic lung-airway parameters

**Table 4:** Comparison of target values of lung volumes, $C_{tot}$ and resistances in literature to model values

	Healthy		Obese		ARDS		COPD		Fibrosis	
	Target	Model [3]	Target	Model	Target	Model	Target	Model	Target	Model
RV (L)	1.2	1.6 ± 0.4	0.5 ± 0.3	0.35 ± 0.05	0.5 ± 0.2	0.044 ± 0.004	f(height,age)	3.3 ± 0.08	1.1 ± 0.2	1.25 ± 0.15
FRC at ZEEP (L)	2.4	3.2 ± 1.2	1.1 ± 0.3	1.3 ± 0.3	1.3 ± 0.3	1.6 ± 0.5	>normal	4.6 ± 0.6	1.6 ± 0.2	1.9 ± 0.2
TLC (L)	6.0	6.7 ± 1.5	5.1 ± 1.5	5.2 ± 1.2	4.8 ± 1	5.4 ± 1.2	2*RV	6.6 ± 0.4	3.4 ± 0.3	3.25 ± 0.15
$R_{insp}$ (cmH2O/L/s)	2 ± 1	3 ± 1	6 ± 2.5	7 ± 1	5 ± 3	6.3 ± 1	7.5 ± 2.5	6.5 ± 2.5	21	2.10.1
$R_{exp}$ (cmH2O/L/s)	2 ± 1	3 ± 1	6 ± 2.5	7 ± 1	5 ± 3	6.3 ± 1	15 ± 5	14 ± 6	2 ± 1	2.1 ± 0.1
$C_{tot}$ at FRC (L/cmH2O)	0.15 ± 0.05	0.15 ± 0.05	0.06 ± 0.05	0.06 ± 0.01	0.045 ± 0.02	0.044 ± 0.004	>0.15	0.165 ± 0.03	0.025 ± 0.01	0.029 ± 0.03

Table 4 shows the target range of $R_{insp}$, $R_{exp}$, $C_{tot}$, and of the lung volumes and the values obtained from the model. The values for the “Healthy” archetype are not tuned but obtained from Athanasiades et al. [3], and therefore differ more from the target values, but are still in the acceptable range. We defined the acceptable range as 0.5 L from the target value for the RV, 1 L for FRC, and 1 L for TLC. For $R_{insp}$ and $R_{exp}$, 1.5 cmH2O/L/s from the target is acceptable, for $C_{tot}$ 0.01 L/cmH2O is.

The data obtained for the other archetypes correspond well to measured airway and lung parameters [2, 4, 7, 9, 17, 18, 21, 29].

### Visual inspection of the waveforms

**Fig. 4:**
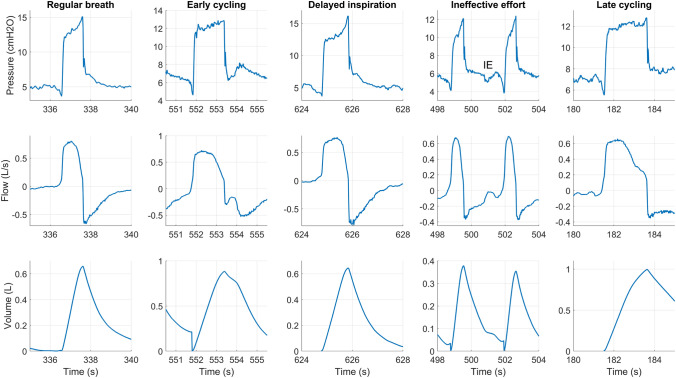
Examples of a regular breath, early cycling, delayed inspiration, ineffective effort (IE), and late cycling in clinical data. The data was recorded after cardiac surgery, no (known) lung dysfunctions were present [13]

Figure 4 shows a normal PSV breath and the four types of asynchronies in clinical data of patients after cardiac surgery (normal lung) [13]. The normal breath shows a rounded peak in the flow during the inspiration phase and a sharp peak in flow during the expiration phase. The pressure increases steeply when the ventilator triggers and the pressure decreases slowly during inspiration until the patient’s muscles relax, after which it increases faster. The flow shows an exponential pattern and the pressure monotonically decreases after cycling. During the early cycling in Fig. 4, the pressure shows a pressure minimum after cycling and characteristic deviation from the exponential flow pattern during expiration, i.e., a strong decrease in expiratory flow directly after cycling. Finally, the volume after the ventilator cycles does not decrease as fast as in the regular breath. Only when the patient stops inspiration the volume decreases at its regular pace. In the delayed inspiration recording in Fig. 4, the patient suffers from cardiac oscillations, causing some extra pressure above PEEP. This causes the ventilator to be late with triggering. Besides the late trigger, the delayed inspiration looks similar to the normal breath. The ineffective effort in Fig. 4 is shown between two regular breaths. During the ineffective effort, a drop in pressure is visible in the pressure at the airway opening, and a small increase in the flow is visible caused by the inspiration effort of the patient without ventilator triggering. During late cycling in Fig. 4 the ventilator cycles later than the patient stops inspiration. This is clearly seen from the shape of the flow during inspiration. The flow first has a rounded shape, but after the inspiration effort end-time the flow decreases in an exponential manner and it fails to reach the cycling threshold quickly. Therefore, the ventilator pressurization lasts longer than the patient’s effort.

**Fig. 5:**
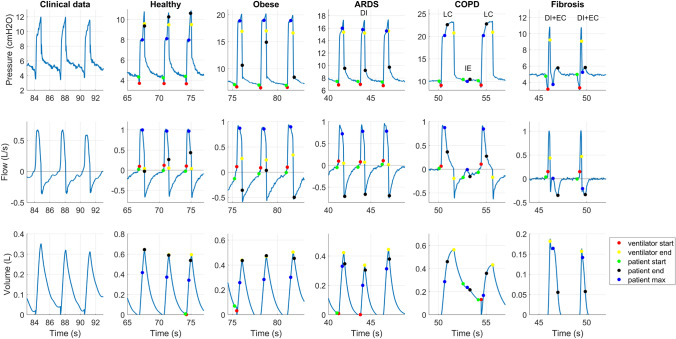
The column on the left shows an example of clinical waveforms with no lung dysfunctions [13]. The five left columns are simulated waveforms for normal, obese, ARDS, COPD and idiopathic fibrosis archetypes. They also show late cycling (LC), ineffective effort (IE), delayed inspiration (DI), and a combination of delayed inspiration-early cycling (DI + EC). The circles in the simulations are the automatically generated labels. The red and yellow, are where the ventilator triggers and cycles. The green is the start of patient inspiration, black the end of patient inspiration, and blue where the patient inspiration is at its maximum

Figure 5 provides a comparison of the clinical data, and the five simulated patient archetypes with different types of asynchronies. The automatically generated labeling is visible in the generated simulated waveforms. In the figure, ineffective efforts, late cycling, delayed inspiration, and a combination of delayed inspiration-late cycling and delayed inspiration-early cycling are visible. They show correspondence to the asynchronies in Fig. 4. The waveforms for the different patient archetypes show the typical characteristics that are expected based on Table 1. The PEEP and $P_{insp}$ are set in such a way that 0.5-L tidal volume was reached, which often corresponds to the clinical target (depending on the bodyweight of the patient). However, due to some asynchronies, this cannot always be reached. Triggering and cycling thresholds were sometimes set slightly too high or too low to generate an asynchrony.

**Fig. 6:**
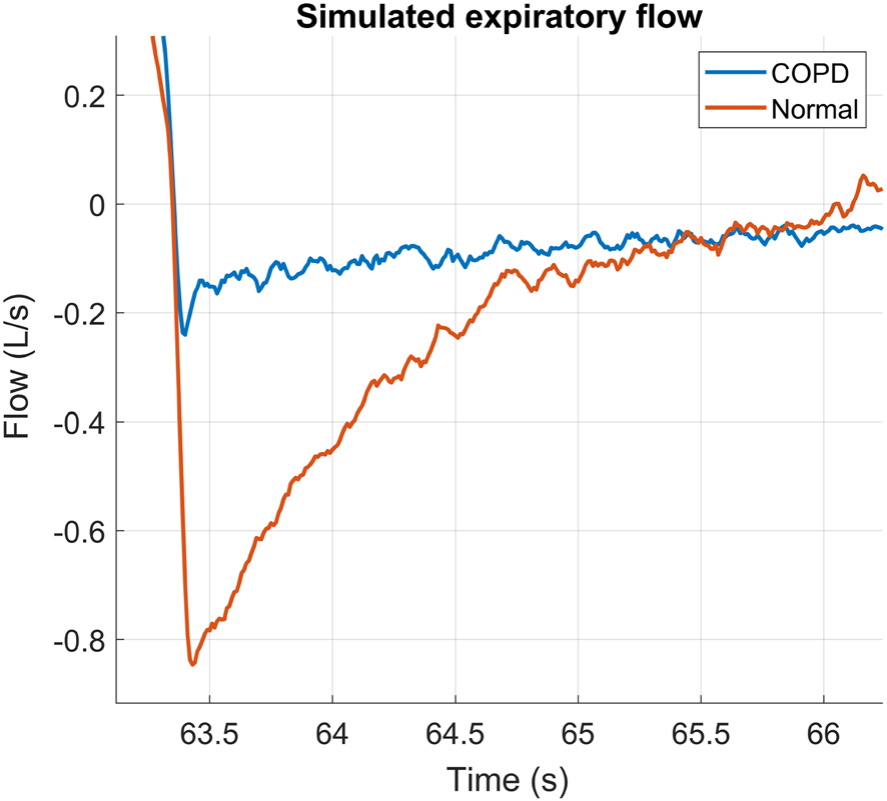
Simulated expiratory flow for a COPD patient and a “normal” (healthy) patient

For COPD and obese patients, expiratory flow limitation is an important feature. Figure 6 shows the differences in the expiratory flows of a COPD patient and a healthy patient in more detail.

**Fig. 7:**
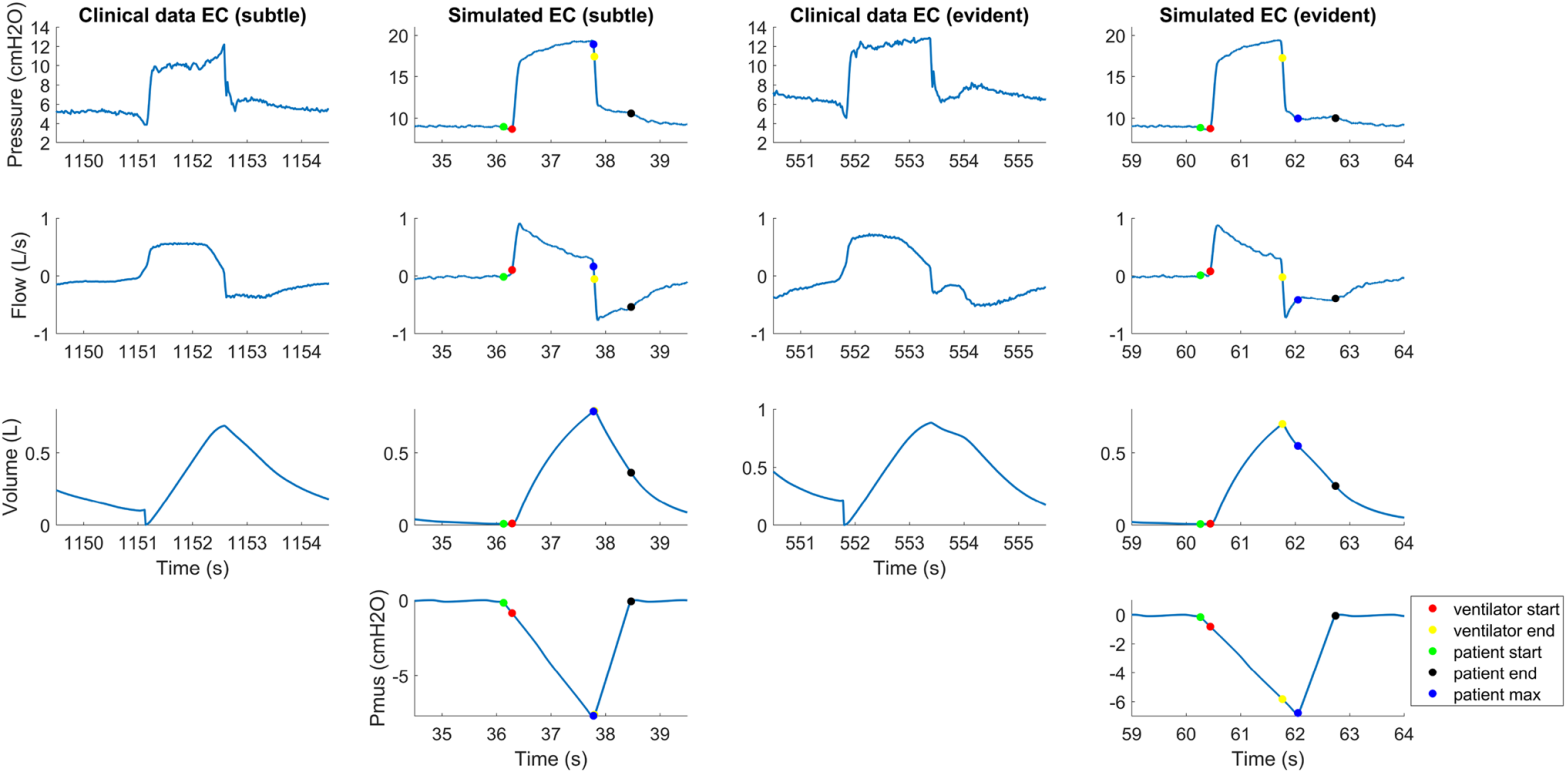
Two breaths with early cycling (EC) in clinical data and two early cycling breaths in simulated data. The simulated waveforms (of the ARDS-archetype) show that the characteristic shape of early cycling is only achieved when the maximum of the patient effort lies after the ventilator end (the two columns on the right). In subtle early cycling, the maximum of patient effort lies before ventilator cycling and the early cycling does not have its characteristic shape (the two columns on the left)

Figure 7 shows an example of mild and evident early cycling in more detail, both in clinical data and in the simulated data. In the case of subtle early cycling, the maximum of patient effort lies just before the point of ventilator cycling. This results in a flattened peak of the expiratory flow, but it does not have the distinct early cycling shape often seen in literature and which is also seen in Figs. 4 and 5. The last two columns show a more severe version of early cycling, where the maximum of the patient effort lies much further beyond the cycling point of the ventilator. This results in the typical early cycling shape with a bump in pressure and in expiratory flow.

In the survey, the response rate of the survey was 87.5%. Only 53% of the recordings were classified correctly as clinical or simulation by the clinicians. The result is not statistically significant (*P* = 0.44, by Fisher’s exact test). This indicates that the clinicians were not able to differentiate between the simulated waveforms and the clinical data.

### Evaluation by machine learning

Table  5 shows the  TPR, PPV, a $\hbox {RMSE}_{\text {start}}$ and $\hbox {RMSE}_{\text {end}}$ of the machine learning algorithm. The overall TPR is 94.3%, while the overall TPR of the algorithm validated on clinical data reported in Bakkes et al. [5] was 98%. The overall PPV on the synthetic data was 93.5%, while on the clinical data this was 98%. The PPV and TPR of the ineffective efforts are lower than average. The PPV is lower than the TPR in case of ineffective efforts since the algorithm identifies segments in the data that are not ineffective efforts (false positives). The $\hbox {RMSE}_\text {end}$ for early cycling is higher than for the other asynchronies.

**Table 5:** Detection results of inspiration effort by the machine learning algorithm

	TPR (%)	PPV (%)	$\hbox {RMSE}_{\mathrm {start}}$ (s)	$\hbox {RMSE}_{\mathrm {end}}$ (s)
Total	94.3	93.5	0.0919	0.120
Delayed inspiration	98.8	100	0.0848	0.154
Early cycling	94.1	100	0.0707	0.259
Late cycling	97.6	100	0.09	0.0519
Ineffective effort	85.9	69.2	0.142	0.0619
Normal	93.5	100	0.0896	0.0826

## Discussion

The simulated waveforms have the advantage that annotation of the start and end of patient effort is available and that unlimited data can be generated.

The original lung model had four parameter sets for healthy lungs. We started by creating more parameter sets to capture the diverse pathologies encountered in the ICU. Table 4 shows that the new parameter values have sufficient correspondence to the target values found in the literature. Slight variations were present when compared to the target values; however, the differences fall into the margins of the allowed patient-to-patient variation and might be postitive for the variation in the dataset.

Figure 5 shows that the simulated waveforms are very close in shape to the clinical waveforms shown in Fig. 4. The model complexity is sufficient to generate waveforms of the correct shape, features, and magnitudes as observed in the clinical data.

In our model, the observed increase in pressure during the end of inspiration is mainly caused by the relaxation of the muscles of the patient and the filling of the lung. During the first phase of inspiration, the patient effort becomes stronger, the difference between the pressure in the lung and the pressure at the airway opening is high, causing a high inspiratory flow. During the second phase, the muscles of the patient relax, and the lung is already partly filled. This leads to a lower pressure difference between the airway opening and the alveolar space, even with the ventilator, which in turn results in a lower inspiratory flow. Since the resistances of the ventilator tubing, endotracheal tube, and the airways are dependent on the flow through turbulence, they become lower.

The pressure drop over the breathing tubes decreases because of the reduced turbulence at reduced flow. This leads to a quickly rising pressure at the airway opening, causing the characteristic peak at the end of inspiration. This increase in pressure can also be attributed to expiratory effort. Although this might often be the case when the peak is observed, in our model no expiratory effort is present. In recent research, it has been shown that it indeed can also be caused by the muscle relaxation at the end of inspiration [33], which supports what we find with the model.

In Fig. 5 the flow has a rounded peak, which is related to patient effort. This is both seen in the clinical data and in the simulated data. After the relaxation of the muscles, the inspiratory flow decays in a passive (exponential) fashion, which is especially visible during late cycling.

The patient archetypes in Fig. 5 show the typical features of each patient archetype. Fibrosis and ARDS show features of patients with shorter time constants: the passive decay of the waveforms is faster than the other archetypes. COPD and the obese archetype have more autoPEEP present than the other archetypes, which is an indication of longer time constants. Figure 6 shows the expiratory flow in more detail. Expiratory flow limitation (EFL) is an important feature of COPD patients and is sometimes also observed in obese patients. In Fig. 6, EFL is clearly seen in the COPD archetype: first, the expiratory flow increases fast, after which there is a much slower decrease caused by rapid change in transmural pressure over the collapsible airways during expiration.

The waveforms of the asynchronies in the simulated waveforms (Fig. 5) are similar to the asynchronies in the clinical data (Fig. 4). In the simulations, ineffective effort, late cycling, and delayed inspiration emerged more often in obese and COPD archetypes. Due to autoPEEP, the trigger threshold is sometimes not reached or is eventually reached resulting in a delayed inspiration. Early cycling was more often observed in ARDS and fibrosis archetypes, which corresponds to clinical observations [11]. In Fig. 7, two examples of early cycling are shown in clinical data and in the simulations. The first two columns show an example of subtle early cycling. In the simulations and in the clinical example, the maximum of the patient effort (patient max) lies before the cycling point of the ventilator. This results in early cycling without its characteristic shape that is often seen in the literature. The early cycling asynchrony can only be recognized by the flattened expiratory peak flow. The two columns on the right of Fig. 7 show a more characteristic early cycling shape. The maximum effort lies after the cycling point of the ventilator, which causes the characteristic shape in the expiratory flow. This is both seen in the simulated data and in the clinical data.

After this visual inspection, the randomly selected clinical and simulated waveforms were given to the clinicians during the survey. The clinicians were not able to significantly distinguish between the simulated waveforms and the clinical waveforms.

The machine learning algorithm trained on clinical data was able to recognize 94% of the patient inspirations in the simulated data. When the clinical dataset was used for validation, the machine learning algorithm recognized 98% of the inspirations [5]. It is always expected that the performance is higher when trained and validated on the same dataset (as is the case for the clinical data) versus an external dataset. The decrease of the PPV and TPR are in line with what would be expected, if one would compare an external dataset with a dataset that is used for training. The simulated data also included more patient archetypes and more subtle asychronies that were on the border between a regular breath and asynchrony, which might have been challenging for the machine learning algorithm since they were not included in the clinical dataset.

The TPR for the ineffective efforts is lower, and the RMSE of the end inspiration for early cycling is larger than for the other asynchronies. The lower detection rate for ineffective efforts may be caused by a smaller inspiration effort in the simulations than in clinical data. Bakkes et al. [5] report that also in clinical data, the detection of ineffective efforts was the most difficult. It is likely that this is a difficult asynchrony for the machine learning to detect, since during the survey and inspection of the ineffective efforts, no anomalies were found.

For early cycling, the high RMSE in the detection of the end inspiration can be explained by the subtle and evident early cycling in Fig. 7. In the case of evident early cycling, the algorithm was able to identify the end inspiration correctly. In the case of subtle early cycling, the algorithm was not able to place the end inspiration point at the exact time, causing a high RMSE. Upon inspection of the clinical training set, this type of subtle early cycling was not often present in the training data. This suggests that including the simulations in the training set could improve the detection of subtle early cycling.

### Limitations of the study

This study has some limitations.

1. The non-linear one lung model is a major simplification of the complex and heterogenous lung and airways of patients with ARDS, COPD, lung fibrosis. Furthermore the parameters of this model were hand tuned and may deviate from actual values. A much more complicated multi compartment model with complex tissue models is needed when more detail is required. However, in this case many more parameters are needed, these are not available and computation effort will increase strongly. We have chosen for a limited model complexity, i.e. to leave out complexities until the simulated data starts to deviate substantially from clinical data for the different patient archetypes. It was found that with the present model the simulated parameters and waveforms resemble those observed in clinical data and are of sufficient quality for the present study.
2. The muscle pressure waveform used is semi-empirical and the range of muscle pressure wave parameters used is limited. The model and parameters used are in line with several clinical studies but improvements may be needed. This more diverse muscle pressure waveform can be added if needed.
3. The model for the ventilator is overly simplistic, the complex hardware and servo control loops in such devices certainly will influence the triggering and cycling time and the waveforms. There is a large difference in devices from different suppliers. The focus of the ventilator model is on the breathing set tube impedance and impact of turbulence on flow resistance. Please note that turbulence in the tubes of the breathing set and endotracheal tube has a large impact on flow resistance of these tubes, can lead to very large flow resistance and generates large pressure and flow noise. Unfortunately the degree of turbulence depends on many subtle aspects. For example on the inspiratory valve properties, bends and kinks in the tubes and the use of humidification and bacterial filters. Such effects were not taken into account. Furthermore the presence of secretions was not taken into account. It would have been better to use measured flow resistance of the tube during measurements of the waveform in ventilated patient, this requires clinical studies and was outside the scope of this work. However, the main features of the pressure waveforms are reproduced well and this supports the present first order ventilator model. It was not deemed necessary to add model complexity as the aim was to strive for minimum model complexity while still be able to generate realistic data.
4. The simulations were done with LTSpice, an open source circuit simulator that is available free of charge at the website from Analog Devices. It is possible that the use of this type of simulator is seen as a limitation. It is less common in the physiological society to use these type of software packages. However, LTSpice is well suited for simulations of active circuits as well as non-linear lumped element models. The package is used on a large scale by customers of a major component supplier and by many electrical engineers. Instead of a limitation we consider the use of this simulator as an asset and encourage more wide spread use in the field of physiology modeling.
5. The data set for clinical testing of the machine learning model that was used to evaluate our data was limited in the number of patients and breaths and lacks sufficient diversity in patient archetypes and has only a small proportion of “borderline asynchronies” like mild early cycling or weak ineffective efforts. It is the aim of this study to add this complexity and diversity by adding more and more diverse data.
6. During the classification study by experienced clinicians, clinicians were allowed to participate voluntarily. It could be that there is a self-selection bias. Also, the clinicians were only shown a limited set of the waveforms.
7. The aim of the work is to provide tools and models for a clinical decision system that assists the clinician in real time to provide optimal lung protective ventilations during mechanical ventilation. The machine learning model can be an important part of a real-time clinical decision support system. However, for the classification of asynchronies a second layer of signal processing is needed. This was not part of the present study.

## Conclusion

In this work we developed a patient-ventilator model that was able to generate annotated flow, pressure and volume waveforms during pressure support ventilation.

Our results indicate that it is feasible to generate labeled synthetic ventilator waveforms using a model-based approach. During the visual analysis, the survey including clinicians, and analysis by the machine learning algorithm, the synthetic data has shown considerable similarity to clinical data.

The dataset helped identify possible improvements for the machine learning algorithm, such as improvement of the detection of the end of inspiration time in weak early cycling and detection of weak ineffective efforts. Moreover, the model has given new insights into the mechanisms of patient-ventilator interactions, and might therefore also have significant value for educational purposes.
